# Diagnostic Performance of a Deep Learning-Powered Application for Aortic Dissection Triage Prioritization and Classification

**DOI:** 10.3390/diagnostics14171877

**Published:** 2024-08-27

**Authors:** Vladimir Laletin, Angela Ayobi, Peter D. Chang, Daniel S. Chow, Jennifer E. Soun, Jacqueline C. Junn, Marlene Scudeler, Sarah Quenet, Maxime Tassy, Christophe Avare, Mar Roca-Sogorb, Yasmina Chaibi

**Affiliations:** 1 Avicenna.AI, 375 Avenue du Mistral, 13600 La Ciotat, France; angela.ayobi@avicenna.ai (A.A.); yasmina.chaibi@avicenna.ai (Y.C.); 2 Department of Radiological Sciences, University of California Irvine, Irvine, CA 92697, USA; 3 Center for Artificial Intelligence in Diagnostic Medicine, University of California Irvine, Irvine, CA 92697, USA; 4 Department of Radiology and Imaging Science, Emory University Hospital, Atlanta, GA 30322, USA

**Keywords:** deep learning, medical and biomedical image processing, aortic dissection, AI-based solution for radiology, machine learning diagnostic performance, medical imaging automated analysis, emergency radiology

## Abstract

This multicenter retrospective study evaluated the diagnostic performance of a deep learning (DL)-based application for detecting, classifying, and highlighting suspected aortic dissections (ADs) on chest and thoraco-abdominal CT angiography (CTA) scans. CTA scans from over 200 U.S. and European cities acquired on 52 scanner models from six manufacturers were retrospectively collected and processed by CINA-CHEST (AD) (Avicenna.AI, La Ciotat, France) device. The diagnostic performance of the device was compared with the ground truth established by the majority agreement of three U.S. board-certified radiologists. Furthermore, the DL algorithm’s time to notification was evaluated to demonstrate clinical effectiveness. The study included 1303 CTAs (mean age 58.8 ± 16.4 years old, 46.7% male, 10.5% positive). The device demonstrated a sensitivity of 94.2% [95% CI: 88.8–97.5%] and a specificity of 97.3% [95% CI: 96.2–98.1%]. The application classified positive cases by the AD type with an accuracy of 99.5% [95% CI: 98.9–99.8%] for type A and 97.5 [95% CI: 96.4–98.3%] for type B. The application did not miss any type A cases. The device flagged 32 cases incorrectly, primarily due to acquisition artefacts and aortic pathologies mimicking AD. The mean time to process and notify of potential AD cases was 27.9 ± 8.7 s. This deep learning-based application demonstrated a strong performance in detecting and classifying aortic dissection cases, potentially enabling faster triage of these urgent cases in clinical settings.

## 1. Introduction

Acute aortic syndrome (AAS) represents a spectrum of pathological conditions affecting the thoracic and abdominal aorta. Their prevalence ranges from 0.2% to 0.8%, equating to approximately 2.6–3.5 cases per 100,000 individuals in the general population, with a predilection towards males [1,2]. Aortic dissection (AD) constitutes the predominant subtype within the range of AAS, accounting for 85–95% of cases [3]. AD arises from a tear in the aorta’s intimal and medial layers, facilitating blood ingress into the aortic wall and the formation of a secondary circulating pathway within the aorta known as the false lumen [4]. This condition presents as a critical, life-threatening emergency, with mortality rates increasing by approximately 1–2% per hour within the initial 48 h [1]. In cases where prompt surgical intervention is not undertaken, the mortality rate escalates to 58% [5]. Therefore, expedited diagnosis is pivotal for effective patient management [6].

In response to the severity of AD, the Stanford classification system was developed, distinguishing between type A and type B dissections. Type A primarily involves the ascending aorta, whereas type B is limited to the descending aorta distal to the left subclavian artery [1,2,4]. Type A AD demands urgent surgical intervention to mitigate the risk of life-threatening complications. Conversely, type B AD can often be managed conservatively through pharmacological means, particularly focusing on hypertension control, although close monitoring and potential intervention remain essential for optimal patient outcomes [1,2].

The clinical presentation of aortic dissection exhibits a lack of specificity, which can result in symptoms resembling those of various other medical conditions. This diagnostic ambiguity poses a significant challenge in the accurate identification of aortic dissection, as its manifestations overlap with a diverse range of diagnoses [5]. Misdiagnosis of AD significantly impacts patient survival. Approximately 25% of AD patients receive inappropriate antithrombotic treatment due to initial misdiagnosis, resulting in an almost 50% increase in long-term mortality [7]. Additionally, misdiagnosed patients experience a prolonged interval from symptom onset to surgery (8.6 h) compared to correctly diagnosed patients (5.5 h), further elevating their mortality risk [8]. A systematic literature review demonstrated an AD misdiagnosis rate of 33.8%, indicating that one-third of patients face increased mortality risk solely due to diagnostic inaccuracies [9]. Hence, ensuring accurate diagnostics and AD type classification is imperative for providing appropriate treatment. Importantly, the utilization of prompt and appropriate imaging techniques is associated with enhanced diagnostic accuracy [9].

In recent years, CTA has emerged as the gold standard for assessing aortic dissection, owing to its exceptional precision and effectiveness in identifying this condition [2,3,10]. Despite the high sensitivity and specificity exhibited by CT diagnostics in detecting aortic dissection (AD), the escalating demands within the emergency environment and the onset of fatigue among radiologists could potentially elevate the error rates and prolong the time required for AD diagnosis. Thus, deep learning (DL) tools for AD detection and prioritization have been shown to expedite the evaluation process for radiologists, consequently reducing the time required for clinical decision making [6]. The current investigation sought to scrutinize the efficacy and applicability of a recently introduced and commercially available DL-based tool for AD case prioritization. Specifically, the objectives of the study encompassed an evaluation of the diagnostic accuracy of the evaluated device, its capacity for the detection of aortic dissection types, and its potential to reduce the notification time of AD occurrences. This study aims to contribute valuable insights into the utility of DL technologies in enhancing the efficiency and precision of AD diagnosis and clinical patient management.

## 2. Materials and Methods

### 2.1. DL-Powered Algorithm for AD Detection: Architecture and Training

A commercially available application for AD (FDA approved and CE marked), CINA-CHEST v1.0.2, was provided by Avicenna.AI (La Ciotat, France). In the development of the AD DL-powered application, a two-stage approach was employed. The first algorithm was utilized for the segmentation of the aorta, while the second algorithm was employed for the localization of the AD within it, specifically focusing on the visible intimal flap between the true and false lumens. This methodology was selected due to its effectiveness in maximizing the detection of AD in anatomically consistent regions. Both algorithms were based on convolutional neural networks (CNNs). A hybrid 3D/2D U-Net variant, known for its robust performance in 2D and 3D segmentation tasks and previously published by Chang et al. [11], was used.

Regarding the ground truth used to train the algorithms, the segmentation of the aorta, encompassing both the true and false lumens while excluding necrosis or intramural hematoma, was performed per slice by two expert radiologists. Per slice segmentation of the visible intimal flap between the false and true lumens was conducted, so that the algorithm could target the localization of the AD.

The training dataset consisted of 649 3D CTA studies sourced from 40 different scanner models, representing various manufacturers (55% GE, 20% Siemens, 10% Philips, and 15% Canon), spanning from January 2019 to December 2022. Of these studies, 25% constituted positive cases of aortic dissection, categorized as type A or type B according to the Stanford classification. The age distribution among positive cases was as follows: 8% in the (18–40) age range, 56% in the (41–70) age bracket, and 36% over 70 years old. The cases with confounding conditions, such as thoracic or abdominal aneurysms, intramural hematoma, calcifications, and post-surgery instances (e.g., presence of stents), were sought out to enrich the training dataset.

### 2.2. Data Selection

The retrospective and observational CTA data acquisition for this study spanned from July 2017 to March 2022 and was conducted using multiple clinical sources. All data were anonymized and supplied by two teleradiology networks located in the USA and France, comprising more than 200 cities and 6 CT makers. The data were acquired on 52 different scanner models. Among the received data, CTA cases were consecutively preselected according to the recommended requirements (Table 1). The final dataset consisted of 1303 cases.

### 2.3. Ethical Considerations for Data

In this study, we adhered to stringent data ethics and privacy standards. All data utilized for algorithm training and analysis were anonymized and provided by two teleradiology companies based in the United States and France. The U.S. data were de-identified directly by the teleradiology company in accordance with the HIPAA Privacy Rule, specifically 45 CFR § 164.514(e) [12], before being transferred to Avicenna.AI. Additionally, per 45 CFR § 46.101 [13], data where individual subjects could not be identified were exempted from institutional review board (IRB) approval. This exemption was granted based on the criterion that the data posed no risk of identifying individual subjects, thereby supporting our commitment to conducting ethically sound and compliant research.

The European data were de-identified through a provider equipped with an advanced de-identification system, compliant with the General Data Protection Regulation (GDPR) (EU) 2016/679 [14], which outlines the requirements for lawful processing and the protection of personal data. This ensured that all data were rendered non-identifiable, maintaining strict confidentiality and privacy. Informed consent was waived when it was deemed necessary, following national legislation and institutional protocols, before the data were transferred to Avicenna.AI.

### 2.4. The Ground Truth

Two U.S. board-certified expert radiologists with 7 and 6 years of experience in radiology clinical practice independently visually annotated chest and thoraco-abdominal CTAs and determined the cases with suspected ADs. For positive cases, the experts defined the AD type according to the Stanford classification (type A or type B). A third U.S. board-certified expert radiologist with 8 years of experience in clinical radiology practice settled any disagreements. A third U.S. board-certified expert radiologist with 8 years of experience in radiology clinical practice settled any disagreements. The presence or absence of AD and AD classification by type were determined by majority agreement. Hyperacute, acute, subacute, or chronic ADs were all considered positive. The radiologists also reported the observed confounding factors such as thoracic or abdominal aneurysms, intramural hematoma, calcifications, and post-surgery instances (e.g., presence of stents).

### 2.5. Data Processing

The next step entailed processing the same anonymized dataset using the CINA-CHEST (AD) v1.0.2 AI-powered application. The application automatically processed incoming CTAs, displaying notifications of suspected findings (if any) alongside image series information. For cases flagged positive by the application, the AD type (A or B) was also displayed. All results were gathered for assessment. The evaluation was conducted blindly, without access to the results of the U.S. board-certified radiologists. Notifications, AD types (for positive cases), and processing times were recorded for all CTA cases, measured from the end of DICOM reception to positive or negative identification.

### 2.6. Statistical Analysis

The results provided by U.S. board-certified radiologists and those automatically computed by CINA-CHEST (AD) were compared. The sensitivity, specificity, accuracy, and the area under the receiver operating characteristic curve (ROC AUC) were calculated for the complete dataset. The 95% confidence intervals (95% CI) for sensitivity, specificity, and accuracy were determined using the exact binomial distribution test (Clopper–Pearson). Matthew’s correlation coefficient (MCC) was also computed to assess the binary classification quality. Positive and negative predictive values were derived using sensitivity and specificity, accounting for prevalence in the current dataset (10.5%). Subset performance analyses based on imaging acquisition parameters (manufacturer and slice thickness) and patient characteristics (age and sex) were conducted. Moreover, the clinical performance of Stanford classification for AD (type A and type B) was determined. The sensitivity, specificity, and accuracy were computed for each type of AD. Additionally, AD prioritization and triage effectiveness were evaluated based on the standalone per-case processing time of the device (mean ± SD, 95%CI, and median values) for all cases in the database and for true positive cases only. Statistical analyses were performed using MedCalc Statistical Software (MedCalc, v20.015MedCalc Software Ltd., Ostend, Belgium).

## 3. Results

### 3.1. Data Distribution

According to the ground truth, the final dataset included 137 (10.5%) positive and 1166 (89.5%) negative cases. Among 137 positive cases, 63 and 74 aortic dissections were identified as type A and type B AD, respectively. The mean ± SD age of 1303 patients included in the study was 58.8 ± 16.4 y/o (min = 18 y/o and max = 90 y/o). The male and female populations were almost equally distributed (46.7% and 53.3%, respectively). The CTA examinations were conducted using different scanner makers: GE (GE Healthcare, Chicago, IL, USA) (259; 19.9%), Philips (Philips Healthcare, Amsterdam, The Netherlands) (489; 37.5%), Siemens (Siemens Healthinners, Erlangen, Germany) (474; 36.4%), Canon (formerly Toshiba) (Canon Medical Systems Corporation, Otawara, Japan) (76; 5.8%), Hitachi (Hitachi Ltd., Tokyo, Japan) (4; 0.3%), and Philips-Neusoft Medical Systems (PNMS) (Neusoft Medical Systems, Shenyang, China) (1; 0.1%). A total of 456 (35%) scans had slice thickness values lower than 1.5 mm, 629 (48%) were lower than 3 mm, and 218 (17%) were equal to 3 mm (Table 2).

### 3.2. Performance Statistical Results

**Figure 1:**
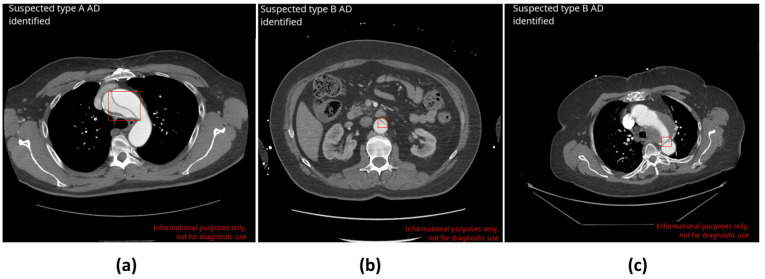
Examples of CINA-CHEST (AD) outputs upon true and false positive AD cases automatically determined by CINA-CHEST (AD). Red boxes are placed by CINA-CHEST (AD) to indicate the localisation of a detected AD. (**a**) Correct detection of type A AD. (**b**) Correct detection of type B AD. (**c**) False-positive identification of a complicated case in the presence of intramural hematoma following aortic repair.

The DL-based application correctly identified 129 AD cases (true positive—TP; Figure 1a,b). Eight cases were missed by the application (false negative—FN), leading to a sensitivity of 94.2% [95% CI: 88.8–97.5%]. In total, 1134 cases were correctly labeled as negative for AD (true negative—TN), and 32 cases were wrongly flagged as positive (false positive—FP; Figure 1c), resulting in a specificity of 97.3% [95% CI: 96.2–98.1%]. Among 1303 scans, no difference was found between the ground truth and AI-based application assessments in 1263 cases, which represents an accuracy of 96.9% [95% CI: 95.8–97.8%]. The area under the receiver operating characteristic curve (ROC AUC) was also calculated: 0.96 [95% CI: 0.95–0.97]. Matthew’s correlation coefficient (MCC) was equal to 0.85. Finally, positive and negative predictive values (PPV and NPV) were derived using the sensitivity, specificity, and prevalence values of the actual dataset (10.5%) and were 80.1% and 99.3%, respectively (Table 3).

**Figure 2:**
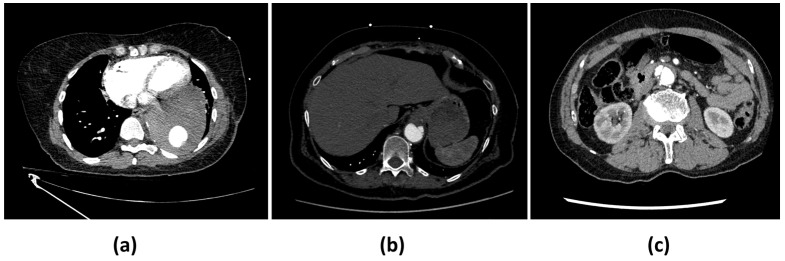
Examples of missed AD cases by CINA-CHEST (AD). (**a**) Missed subtle type B AD due to a streak artefact. (**b**) Missed type B AD in the presence of penetrating atherosclerotic ulcer. (**c**) Missed type B AD in the presence of large intramural hematoma and penetrating atherosclerotic ulcer.

Among the eight missed ADs (FNs), six are complicated cases and were the subject of disagreements between both truthers. Thus, even visually, the detection of these aortic dissections was not obvious. Four of these dissections were located within the abdominal infrarenal aorta or the distal descending thoracic aorta. They were all related to AD type B. Among the confounding factors that impacted correct AD identification by the AI-based algorithm was the combination of acquisition artefacts (one scan was noisy, one presented streak artefacts, and one included motion artefacts) and aortic pathologies (intramural hematoma (IMH): four cases; penetrating atherosclerotic ulcer (PAU): two cases; aortic calcifications: two cases; one aneurysm with large mural thrombus; one endoleak with active extravasation of contrast from the graft in the mid-descending thoracic aorta). One additional case was missed due to bad contrast filling. The last scan presented AD only within the last two abdominal slices, since the acquisition stopped at the level of the right kidney, thus preventing the visualization of the entire dissection (Figure 2; for more details, see Supplementary Table S1).

There were 32 false positive cases. The inaccurate identification of the dissections, resulting in false positives, stemmed from various factors including inadequate contrast opacification (13 cases), motion artefacts (10 cases), instances of pathology mimicking dissection (i.e., penetrating atherosclerotic ulcer (PAU), intramural hematoma (IMH), and aneurysms (7 cases)), and interference from stent grafts (2 cases).

The reasons for misdiagnoses (FN and FP) are summarized in Table 4.

### 3.3. Stanford AD Type Classification

In the current dataset, the ground truth identified 63 positive AD cases as type A dissections and 74 as type B. Among the 137 positive cases, the AI-based application accurately classified all type A dissections and generated 7 false positives, resulting in a sensitivity of 100% [95% CI: 92.8–00%] and a specificity of 99.4% [95% CI: 98.8–9.8%]. For type B AD, the AI-based application produced 8 false negatives and 25 false positives, yielding a sensitivity of 89.2% [95% CI: 79.3–4.9%] and a specificity of 97.9% [95% CI: 97.0–8.7%] (Table 5). The application classified positive cases by AD types with an accuracy of 99.5% [95% CI: 98.9–99.8%] for type A and 97.5 [95% CI: 96.4–98.3%] for type B (Table 5).

### 3.4. Stratified Statistical Analysis Results

The stratified statistical analyses are provided for the subgroups of the CT-scan makers, image acquisition (slice thickness), age, and sex groups (Table 6). The AI-based algorithm performances across these groups are presented in Table 6. The in-depth stratified statistical analysis revealed sensitivities ranging from 89.2% to 100% and specificities from 96.2% to 100%. This comprehensive examination demonstrated that across all categories and within each group, both the sensitivities and specificities consistently surpassed the 89% threshold, and the accuracy for all groups was higher than 95%.

### 3.5. Time to Notification Evaluation Results

The application was run with the following hardware specifications: CPU: 8 threads/16 cores at 3.0+ GHz and RAM: 16 GB on Ubuntu 22.04.4 LTS. The time to notification (TTN) was calculated for all 1303 cases. The mean TTN ± SD was 27.9 ± 8.7 s, 95% CI 27.4–28.3 s. The median TTN value was 26.7 s. The mean TTN ± SD for 129 true positive cases was 35.4 ± 15.4 s, 95% CI 30.8–36.3 s, with a median of 33.3 s.

## 4. Discussion

A retrospective, multinational, multicenter, multiscanner blinded study was conducted to evaluate the diagnostic accuracy of the deep learning (DL)-based application CINA-CHEST (Avicenna.AI, La Ciotat, France) for prioritization and triage of aortic dissection (AD) on CTA cases. The database represented a large number of negative AD scans in order to approach the prevalence found in clinical routine. Among the included 1303 CTA scans, 137 (10.5%) cases were positive for AD, as established by the majority agreement of three U.S.-board certified expert radiologists. The DL-based application correctly labeled 129 cases as positives and 1134 as negatives, yielding 8 false negatives and 32 false positives. Therefore, the specificity and sensitivity of the application were 94.2% [95%CI: 88.8–97.5%] and 97.3% [95%CI: 96.2–98.1%], respectively. The application correctly classified the type of dissection with a sensitivity and specificity of 100% and 99.4% for type A and 89.2% and 97.9% for type B, respectively. The main causes of misdiagnoses were mainly pathologies mimicking dissection and acquisition artefacts. Moreover, the mean time to notification for all cases in the current dataset was evaluated (27.9 s) and was compatible with the practical use in emergency radiology.

Therefore, the assessed deep learning application successfully conducted screening (triage), classification (type A and type B dissection), and prioritization (operator notification) for the presence of suspected aortic dissections (AD). Several studies in the literature have explored DL-based algorithms aimed at detecting AD and assessed the same parameters (Table 7).

Enhancing the diagnostic capacity of radiologists’ AD screenings using non-enhanced CT scans was the objective of two studies [15,17]. The diagnostic performances of the DL-based algorithms used were similar to or slightly outperformed those of trained radiologists. Hata et al. [15] showed that the sensitivity and specificity of their DL-based application were 91.8% and 88.2%, whereas trained radiologists performed at 90.6% and 94.1%, respectively. Yi et al. [17] sought to improve upon the DL-model implemented by Hata et al. [15] and developed a deep integrated model with a sensitivity and specificity of 86.2% and 92.3%, respectively. However, passing their DL-based algorithm on cases obtained from an external clinical center drastically dropped the specificity to 55.4%. These findings emphasize the importance of cases originating from different clinical sources, CT scan makers, and acquisition parameters for a proper diagnostic performance evaluation. Moreover, both studies mentioned above were conducted with a high prevalence of positive AD cases, which does not occur in clinical routine.

Harris et al. [6] evaluated their DL-based tool for AD detection using a multicenter, multiscanner approach using CTA images with a low AD prevalence. The sensitivity and specificity of this application were 87.8% and 96.0%, respectively. In comparison, CINA-CHEST (AD) (Avicenna.AI, La Ciotat, France) underwent an evaluation using scans sourced from multiple clinical sources, various scanner makers and models, and diverse acquisition parameters and approaches to real-world disease prevalence. Therefore, the current performance evaluation, conducted under conditions closely resembling real-world clinical practice, showcased a superior diagnostic performance to previously published solutions for AD triage and prioritization. Similar to CINA-CHEST (AD), another deep learning solution for the triage and prioritization of AD, Briefcase for AD (Aidoc Medical, Tel Aviv, ISRAEL), has received regulatory clearance and certification. Briefcase for AD demonstrated a sensitivity of 93.23% (95% CI: 88.70–6.35%) and a specificity of 92.83% (95% CI: 89.35–5.45%) on 499 CTAs cases, including 192 positives. Therefore, CINA-CHEST (AD) outperformed this similar solution [18]. Additionally, CINA-CHEST (AD) is unique among certified and commercially available applications in its ability to classify aortic dissection by Stanford classification types.

AD type identification is a crucial feature of AD screening DL-based applications, as it allows clinicians to promptly sort patients in the emergent surgery clinical pipeline (for type A) and conservative treatment pipeline (for type B). Deploying a two-step neural network, Huang et al. [16] demonstrated the capacity of a DL-based application to classify AD by type with a sensitivity and specificity of 95.5% and 98.5% for type A and 79.3% and 94.0% for type B. In line with previously published articles, CINA-CHEST (AD) successfully identified all type A AD cases. All eight ADs missed by the application were type B, demonstrating more complicated automated diagnostics for these cases. In fact, as stated by Yi et al. [17], the diagnostic performance for type B is shown to be lower than for type A. This is due to a wider range of dissections, as a larger relative aorta volume is found in the descending aorta than in ascending aorta. However, this does not adversely affect clinical outcomes because type A ADs are life-threatening and require immediate intervention, making the accurate detection of these cases significantly more critical.

Early surgical intervention for type A aortic dissection (AD) significantly reduces mortality, emphasizing the crucial need to minimize diagnostic delays [19]. Nevertheless, diagnostic delays are observed in nearly 25% of cases [19]. The reason for these delays is the in-hospital diagnostic times, which are twice as long as the hospital arrival times, contributing to the majority of the delay [20]. The analysis of the International Registry of Acute Aortic Dissection revealed a median diagnostic time of 4.3 h for this acute condition, indicating a significant opportunity for improvement [21]. Diagnostic delays are associated not only with misdiagnosis but also with factors such as physician workload, knowledge, and experience, especially when AD presents without obvious clinical manifestations [22]. Younger patients with AD experience longer diagnostic delays due to clinical hesitations and a lack of suspicion among emergency clinicians [23]. Although CT scans are employed to confirm the diagnosis prior to surgery, their use is linked to diagnostic delays that need to be addressed [24,25]. Implementing a decision support system, such as an automated detection and prioritization application, could enhance clinical workflows by reducing both the misdiagnosis rates and diagnostic delays [26]. Harris et al. [6] measured the notification time of the application from image download into the platform to visible notification of the application results. This time was equal to 23.5 ± 21 s. Images flagged as positive were prioritized for readers’ evaluation. This prioritization impacted the time of delay (time from the image receiving and image opening for analysis). Cases flagged as positive had a significantly reduced median delay time (265 s against 660 s). Considering that according to this study, acute cases must have been addressed within 30 min, the improvement in reducing delays was 20%, representing a significant advancement in the context of emergency radiology [6]. CINA-CHEST (AD) had a similar notification time of 27.9 ± 8.9 s. A similar solution, Briefcase for AD (Aidoc Medical, Tel Aviv, ISRAEL), demonstrated a mean notification time of 38 s [18]. Although CINA-CHEST (AD) has not yet been evaluated in clinical settings, it is hypothesized that the proposed automated solution for AD detection and prioritization could provide substantial clinical benefits by reducing misdiagnoses and diagnostic delay times.

The evaluated application CINA-CHEST (AD) generated a few false positive and false negative cases. The causes of these misdiagnoses were mainly the pathologies mimicking the aortic dissection, like a penetrating atherosclerotic ulcer (PAU), intramural hematoma (IMH), aneurysms, and acquisition artefacts. For all false positive cases, an alert will be dispatched to the on-call clinician, granting them the opportunity to examine the images and offer the appropriate diagnosis. For all misdiagnosed positive cases, as no notification will be issued to the clinician, these cases will instead undergo review via the standard care workflow, ensuring accurate diagnosis by the physician.

Our study had some limitations. Primarily, it did not include a direct comparison between the performance of the deep learning algorithm and that of a panel of independent radiologists. Hata et al. [15] and Yi et al. [17] assessed the radiologist’s performance for AD detection; however, as this evaluation was performed on non-enhanced CT scans, this information is not relevant for CTA images. Second, the retrospective selection of CTA cases may introduce selection bias. Prospective studies would not only demonstrate a real-world performance regarding optimal and sub-optimal CTA scans but would also reveal the supposed clinical benefits regarding time savings for diagnosis in such acute conditions as AD. Moreover, radiologists’ time savings by automated triage applications might become a crucial benefit in the next few years, as the clinician workforce is limited under an ever-increasing workload [27]. Finally, we did not include any clinical parameters in the diagnostic pipeline like Yi et al. [17], who presented an integrated model where aorta morphology was taken into account for AD triage.

## 5. Conclusions

To sum up, CINA-CHEST (AD) (Avicenna.AI, La Ciotat) is a DL-based application for performing triage, classification, and prioritization of aortic dissections. Our multinational, multicenter, multiscanner study demonstrated the highest diagnostic performance reported in the literature for this class of devices. The study was performed with a prevalence that approaches to real-world clinical data, and the dataset presented a significant distribution among clinical sites, scan vendors, and acquisition parameters. This illustrates the device’s robustness for extensive use across varied datasets and patient demographics. Moreover, the clinical use of this application is associated with a prompt time to notification that may improve the diagnostic speed and accuracy of clinicians in exigent emergency settings.

## Figures and Tables

**Table 1:** CINA-CHEST (AD) inclusion and exclusion criteria.

**The Inclusion Criteria for CINA-CHEST (AD)**
Chest or thoraco-abdominal CTA scans Age ≥ 18 y/o Matrix size ≥ 512 × 512 (rectangular matrix accepted) Axial acquisition only Slice thickness ≤ 3 mm with no gap between successive slices Radiation dose parameters: 60 kVp to 160 kVp Reconstruction diameter above 200 mm Density threshold in the aorta ≥ 140 HU Soft tissue reconstruction kernel Field of view including the aortic arch and thoracic aorta
**The Exclusion Criteria for CINA-CHEST (AD)**
Parameters not compatible with acquisition protocol Thoracic aorta out of the field of view Significant motion artefacts (uninterpretable images) Significant streak artefacts (uninterpretable images) Significant noise (uninterpretable images) Bad bolus timing (uninterpretable images)

**Table 2:** The dataset characteristics.

Characteristic	Parameters	AD Dataset (1303 Cases)	AD Positive Cases (137 Cases)
Age	Mean ± SD	58.8 ± 16.4 y/o	59.0 ± 13.3 y/o
Sex	Male	609 (46.7%)	84 (61.3%)
	Female	692 (53.3%)	53 (38.7%)
Scanner makers	GE	259 (19.9%)	77 (56.2%)
	Philips	489 (37.5%)	14 (10.2%)
	Siemens	474 (36.4%)	33 (24.1%)
	Canon	76 (5.8%)	13 (9.5%)
	Hitachi	4 (0.3%)	0 (0.0%)
	PNMS	1 (0.1%)	0 (0.0%)
Slice thickness	<1.5 mm	456 (35%)	53 (38.7%)
	1.5 mm < ST < 3 mm	629 (48%)	56 (40.9%)
	=3 mm	218 (17%)	28 (20.4%)

**Table 3:** CINA-CHEST (AD) confusion matrix and performance data. (TP—true positive, TN—true negative, FP—false positive, and FN—false negative).

Confusion Matrix		Ground Truth		
		Positive	Negative	Total
CINA-CHEST (AD) *	Positive	129 (TP)	8 (FN)	137
	Negative	32 (FP)	1134 (TN)	1166
	Total	161	1142	1303

* Sensitivity [95% CI], %: 94.2 [88.8–97.5]; specificity [95% CI], %: 97.3 [96.2–98.1]; accuracy [95% CI], %: 96.9 [95.8–97.8]; ROC AUC [95% CI]: 0.96 [0.95–0.97]; MCC: 0.85; PPV: 80.1%; NPV: 99.3%.

**Table 4:** Reasons for AD misdiagnoses by CINA-CHEST (AD).

Main Reasons for False Negatives (*n* = 8)	Main Reasons for False Positives (*n* = 32)
Intramural hematoma (IMH) (4)	Inadequate contrast opacification (13)
Penetrating atherosclerotic ulcer (PAU) (2)	Motion artefacts (10)
Acquisition artefacts (2)	Instances of pathology mimicking dissection (7)
	Interference from stent grafts (2)

**Table 5:** Stanford AD classification performances of CINA-CHEST (AD) application. (TP—true positive, TN—true negative, FP—false positive, and FN—false negative).

AD Type	Sensitivity [95% CI], %	Specificity [95% CI], %	Accuracy [95% CI], %
Type A	100 [92.8–100] (TP = 63; FN = 0)	99.4 [98.8–99.8] (TN = 1233; FP = 7)	99.5 [98.9–99.8]
Type B	89.2 [79.3–94.9] (TP = 66; FN = 8)	97.9 [97.0–98.7] (TN = 1204; FP = 25)	97.5 [96.4–98.3]

**Table 6:** Detailed stratified statistical analysis of CINA-CHEST (AD) application.

Parameter	Condition	Sensitivity [95% CI], %	Specificity [95% CI], %	Accuracy [95% CI], %
Age	18 ≤ Age < 40	100 [47.8–100]	97.7 [94.3–99.4]	97.9 [94.5–99.4]
	40 ≤ Age ≤ 60	97.1 [89.9–99.6]	98.2 [96.3–99.3]	98.0 [96.3–99.1]
	Age > 60	90.5 [80.4–96.4]	96.5 [94.7–97.8]	95.9 [94.1–97.3]
Sex	Male	96.4 [89.9–99.2]	96.9 [95.1–98.3]	96.9 [95.1–98.1]
	Female	90.6 [79.3–96.9]	97.5 [95.9–98.6]	96.9 [95.4–98.1]
Scanner makers *	GE	94.8 [87.2–98.6]	96.2 [92.2–98.4]	95.8 [92.5–97.9]
	Philips	92.2 [66.1–99.8]	96.8 [94.8–98.2]	96.7 [94.6–98.1]
	Siemens	93.9 [79.8–99.3]	97.7 [95.8–98.9]	97.5 [95.6–98.7]
	Canon	93.3 [62.1–99.6]	100 [92.8–100]	98.7 [92.9–99.9]
Slice thickness	<1.5 mm	90.5 [79.3–96.9]	97.7 [95.8–98.9]	96.9 [94.9–98.3]
	1.5 mm < ST < 3 mm	98.2 [90.5–99.9]	96.7 [94.9–98.0]	96.8 [95.1–98.0]
	=3 mm	92.9 [76.5–99.1]	97.8 [94.7–99.4]	97.2 [94.1–99.0]

* No stratification was conducted for PNMS and Hitachi Ltd. scanner makers, as a small number of scans were included with these scanners, 1 and 4 CT scans, respectively.

**Table 7:** Summary of previously published studies pertaining to automated detection solutions for AD.

Parameter	Harris et al., 2019 [6]	Hata et al., 2020 [15]	Huang et al., 2022 [16]	Yi et al., 2022 [17]	Current Study
Image type	CTA	Non-enhanced CT	CTA	Non-enhanced CT	CTA
Architecture	5-layer CNN	CNN Xception	2-step network: attention U-net and ResNeXt	Deep integrated model: 2.5D U-net, ResNet34	2-step 2.5D U-Net: aorta isolation and dissection detection
Model	2D	2D	3D	3D	3D
Population	34,577 cases (112 AD pos)	170 cases (85 AD pos)	298 cases (51 pos: 22 type A; 29 type B)	452 cases (internal cohort (341): 139 AD pos. external cohort (111): 46 AD pos.)	1303 cases (137 AD pos)
Enrolment	Retrospective	Retrospective	Retrospective	Retrospective	Retrospective
Samples	Multicenter and multiscanner	One center	One center	Internal center and external center	Multicenter, multiscanner, and multinational
Sensitivity	87.8%	91.8%	Type A: 95.5% Type B: 79.3%	Internal: 86.2% External: 97.8%	All: 94.2% Type A: 100% Type B: 89.2%
Specificity	96.0%	88.2%	Type A: 98.5% Type B: 94.0%	Internal: 92.3% External: 55.4%	All: 97.3% Type A: 99.4% Type B: 97.9%
Features	Triage Mean time to notification: 23.5 ± 21.0 [SD] seconds	Triage Comparison with experts (5 readers): Sensitivity: 90.6% Specificity: 94.1%	Type A/B classification	Triage Comparison with experts (3 readers): Internal experts: Mean sensitivity: 72.7% Mean specificity: 98.3% External experts: Mean sensitivity: 40.6% Mean specificity: 94.0%	Triage Type A/B classification Mean time to notification: 27.9 ± 8.2 [SD] seconds

pos—positive cases.

## Data Availability

The study data are the property of Avicenna.AI and are not publicly accessible. They can be obtained from the corresponding author upon reasonable request and with the approval of the Regulatory Affairs Department of Avicenna.AI.

## Supplementary Materials

The following supporting information can be downloaded at: https://www.mdpi.com/article/10.3390/diagnostics14171877/s1, Table S1: Summary of ADs missed by the DL-based application.
