# Supplementary material for: Diagnostic Performance of a Deep Learning-Powered Application for Aortic Dissection Triage Prioritization and Classification
Source: Diagnostics (Basel). 2024 Aug 27;14(17):1877. doi: 10.3390/diagnostics14171877 (PMC11393899; doi:10.3390/diagnostics14171877)
Supplement: Supplementary file 1 [file diagnostics-14-01877-s001.zip › diagnostics-3122355-supplementary.pdf]

Article: Diagnostic Performance of a Deep Learning-Powered Application for Aortic Dissection Triage Prioritization and Classification.

Supplementary Table S1. Summary of missed ADs by the DL-based application.

FN Case	*Possible reason for misdiagnosis*
1	*Subtle calcified abdominal infrarenal AD was in the presence of very noisy images and intramural hematoma (IMH) "around" the dissection*
2	*AD only within the last most inferior two abdominal slices since the acquisition stopped at the level of the right kidney; therefore, the entire dissection was not visible*
3	*Diffuse IMH involving the entire descending thoracic aorta and a Penetrating Atherosclerotic Ulcer (PAU)*
4	*A missed case showed the presence of a PAU*
5	*IMH involving the descending thoracic aorta from the level of the takeoff of the left subclavian artery*
6	*IMH and thoracic and abdominal aortic aneurysms with large mural thrombus*
7	*Streak artefact due to the placement of a graft beginning at the aortic arch and extending down into the upper abdomen and an endoleak with active extravasation of contrast from the graft in the mid-descending thoracic aorta*
8	*AD was located within motion artefacts, calcified aorta (calcifications are seen around the true lumen), and suboptimal contrast bolus timing*
